# Characterization of bortezomib-adapted I-45 mesothelioma cells

**DOI:** 10.1186/1476-4598-9-110

**Published:** 2010-05-18

**Authors:** Lidong Zhang, James E Littlejohn, Yu Cui, Xiaobo Cao, Chander Peddaboina, W Roy Smythe

**Affiliations:** 1 Section of Surgery Research, Department of Surgery, Texas A & M University Health Science Center College of Medicine and Scott & White Memorial Hospital, Temple, Texas, USA; 2 Department of Oncology, Tianjin Union Medicine Center, Tianjin, China

## Abstract

**Background:**

Bortezomib, a proteasome-specific inhibitor, has emerged as a promising cancer therapeutic agent. However, development of resistance to bortezomib may pose a challenge to effective anticancer therapy. Therefore, characterization of cellular mechanisms involved in bortezomib resistance and development of effective strategies to overcome this resistance represent important steps in the advancement of bortezomib-mediated cancer therapy.

**Results:**

The present study reports the development of I-45-BTZ-R, a bortezomib-resistant cell line, from the bortezomib-sensitive mesothelioma cell line I-45. I-45-BTZ-R cells showed no cross-resistance to the chemotherapeutic drugs cisplatin, 5-fluorouracil, and doxorubicin. Moreover, the bortezomib-adapted I-45-BTZ-R cells had decreased growth kinemics and did not over express proteasome subunit β5 (PSMB5) as compared to parental I-45 cells. I-45-BTZ-R cells and parental I-45 cells showed similar inhibition of proteasome activity, but I-45-BTZ-R cells exhibited much less accumulation of ubiquitinated proteins following exposure to 40 nm bortezomib. Further studies revealed that relatively low doses of bortezomib did not induce an unfolded protein response (UPR) in the bortezomib-adapted cells, while higher doses induced UPR with concomitant cell death, as evidenced by higher expression of the mitochondrial chaperone protein Bip and the endoplasmic reticulum (ER) stress-related pro-apoptotic protein CHOP. In addition, bortezomib exposure did not induce the accumulation of the pro-apoptotic proteins p53, Mcl-1S, and noxa in the bortezomib-adapted cells.

**Conclusion:**

These results suggest that UPR evasion, together with reduced pro-apoptotic gene induction, accounts for bortezomib resistance in the bortezomib-adapted mesothelioma cell line I-45-BTZ-R.

## Background

The 26S proteasome is a multi-subunit enzymatic complex composed of a barrel-shaped 20S core region with catalytic activity adjacent to a 19S regulatory complex [1]. Recent investigations have revealed that the ubiquitin-proteasome pathway plays a key role in regulating the homeostasis of cellular proteins involved in cell cycle regulation, cell survival, and apoptosis. Therapeutic targeting of the proteasome pathway with the specific inhibitor bortezomib has been successful in selectively inducing apoptosis in mesothelioma and a variety of other human cancer cells, with tolerable toxicity to normal cells and tissues [2-4]. Importantly, bortezomib has received US FDA approval for the treatment of patients with multiple myeloma (MM) and mantle cell lymphoma [5].

However, cancer cell resistance to bortezomib-mediated apoptosis may limit the successful application of bortezomib as a cancer therapeutic agent. Although bortezomib shows much stronger anti-tumor activity in MM than in solid tumors, approximately 50% of MMs do not respond to this medication [6,7]. Moreover, many patients with MM who initially responded to bortezomib ultimately relapse with bortezomib-refractory disease [8], suggesting that even in the cancer exhibiting the best treatment response, bortezomib resistance remains a significant obstacle to treatment efficacy.

Bortezomib (PS-341, or Velcade) is a dipeptidyl boronic acid that reversely inhibits 20S proteasome activity. In MM, the transcriptional regulatory protein nuclear factor κB (NFκB) has been proposed as a major target of bortezomib [4,9,10]. Bortezomib blocks the degradation of IκB, a cytoplasmic NFκB inhibitory protein, effectively reducing NFκB translocation from the cytoplasm to the nucleus and blocking its transcriptional regulatory activity. Given the established roles of NFκB in angiogenesis, cell invasion, oncogenesis, proliferation, and inhibition of apoptosis, inhibition of this important transcription factor is widely regarded as an attractive strategy of cancer therapy and a primary mechanism of bortezomib anti-tumor activity in MM cells [4,9,10]. Moreover, as a proteasome inhibitor, bortezomib is able to overcome chemoresistance or induce chemosensitization by inhibiting the NFκB functions that are typically activated by conventional chemotherapeutic agents [9,10]. Beyond NFκB inhibition, bortezomib also induces the intracellular unfolded protein response (UPR) [11] and stabilizes the expression of the proapoptotic genes p53 [12], Bim [13], or noxa [14], indirectly contributing to bortezomib anti-tumor activity.

Although progress has made in defining bortezomib mechanisms of action, mechanisms of bortezomib resistance in cancer are not well understood. In one early study, heat shock protein 27 (HSP27) was shown to play an important role in bortezomib resistance [15]. Recently, evidence was reported supporting a relationship between proteasome subunit β5 (PSMB5) expression and bortezomib resistance. Bortezomib is a reversible inhibitor that primarily targets PSMB5, which is responsible for the chymotrypsin activity of the 26S proteasome. Several studies focused on acute myeloid leukemia, lymphoma, and MM have shown that a series of bortezomib-adapted cell lines developed from the above malignancies exhibit higher PSMB5 expression at the both RNA and protein levels than the respective parental bortezomib-sensitive cells [16-19]. Further investigation demonstrated that inhibition of PSMB5 expression partially restored bortezomib sensitivity in resistant cells [18].

In the present study, a novel bortezomib resistant cell line was developed from the mesothelioma cell line I-45. Our results suggest that UPR evasion together with reduced pro-apoptotic gene induction accounted for bortezomib resistance in this new bortezomib-adapted mesothelioma cell line.

## Results

### Development of the bortezomib-adapted mesothelioma cell line I-45-BTZ-R

**Figure 1:**
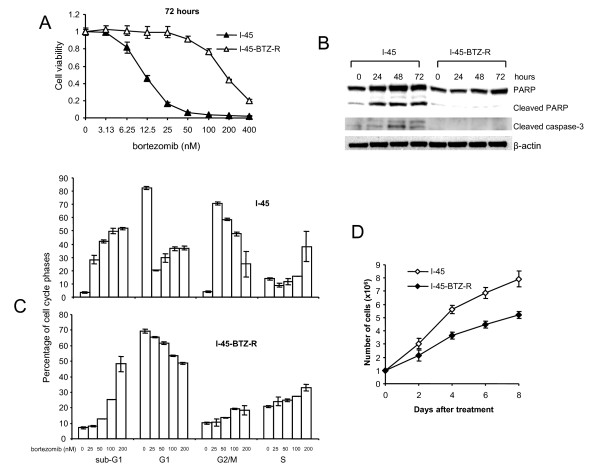
**Characterization of the bortezomib-adapted cell line I-45-BTZ-R**. **A**: I-45 and I-45-BTZ-R cells were treated with bortezomib (3.13 nM to 400 nM) for 72 hours. Cell viability was determined after treatment using the XTT assay. Control cells were treated with PBS and their viability was set as 100%. Values are the mean ± SD of triplicate assays from two experiments. **B**: I-45 and I-45-BTZ-R cells were treated with 40 nM bortezomib for 24, 48, or 72 hours. Caspase-3 and PARP activation (cleavage) were analyzed by Western blot analysis. **C**: I-45 and I-45-BTZ-R cells were treated with bortezomib (25 nM to 200 nM) for 72 hours. Percentages of sub-G1 cells and cell cycle distribution were determined by flow cytometry analysis. Values are the mean ± SD of two experiments. **D**: I-45 and I-45-BTZ-R cells were seeded into 20 cm cell culture dishes (1 × 10^6 ^cells per dish). At days 2, 4, 6, or 8, the cells were trypsinized and stained with trypan blue. Viable cells were counted under a microscope using a hemocytometer. Values are the mean ± SD of three experiments.

To determine whether prolonged exposure of the mesothelioma cell line I-45 to bortezomib would select for cells resistant to bortezomib treatment, I-45 cells were exposed to 12 nM bortezomib (its IC_50 _concentration). The RPMI-1640 cell culture medium was changed every three days and fresh bortezomib was added at the same time. After one month of cell incubation at this concentration, the selection concentration was increased to 15 nM. Extracellular concentrations of bortezomib were increased in a step-wise fashion over a period of 6 months, culminating in a drug concentration of 40 nM. Selection was halted at this dose, since 40 nM is already higher than average plasma concentrations measured in patients following administration of therapeutically effective does of bortezomib. The Bortezomib-adapted mesothelioma cell line I-45-BTZ-R showed consistent resistance to bortezomib, as I-45-BTZ-R cells maintained the same degree of resistance after two months of culture in bortezomib-free medium. As is presented in Figure 1A, three days after treatment with different concentrations of bortezomib, I-45-BTZ-R cells showed much higher cell viability than parental I-45 cells, with IC_50 _values of 185.5 ± 0.3 nM and 12.1 ± 0.4 nM, respectively. Cleavage of caspase-3 or PARP was used as an indicator of bortezomib-induced apoptosis. As seen in Figure 1B, one, two, or three days after cells of exposure to 40 nM bortezomib, this agent induced caspase-3 and PARP cleavage in parental I-45 cells, but not in I-45-BTZ-R cells. These data indicated that I-45-BTZ-R cells do not undergo apoptosis following bortezomib treatment. Cell cycle analysis of treatment-induced sub-G1 cell populations (Figure 1C) confirmed these results. As expected, bortezomib did not induce strong G2/M arrest in I-45-BTZ-R cells as compared with parental I-45 cells (Figure 1C). 25 nM bortezomib treatment for 72 hours increased G2/M population to 70.9% from 4% of the basal level in parental I-45 cells. However, the same treatment even did not change G2/M phase population in I-45-BTZ-R cells, which remained 10.5% as compared to 10.1% of the basal level. With increase in treatment doses, bortezomib induced less G2/M arrest in I-45 cells, which may indicate that more cells at G2/M phase went to apoptotic sub-G1 phase.

To determine cell growth kinetics, the same numbers of both cell types were maintained in culture for eight days. Interestingly, I-45-BTZ-R cells exhibited significantly reduced cell growth as compared to I-45 cells (p < 0.05 at day 2; p < 0.01 at days 4, 6, and 8) (Figure 1D).

### Proteasome subunit PSMB5 protein expression and chymotrypsin-like activity of in I-45-BTZ-R and I-45 cells

**Figure 2:**
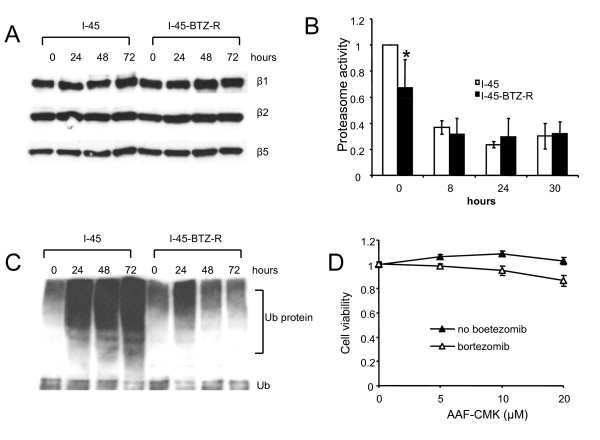
**Expression of proteasome subunit proteins, proteasome activity, and ubiquitinated protein accumulation**. **A**: I-45 and I-45-BTZ-R cells were treated with 40 nM bortezomib for 24, 48, or 72 hours. Expression of the proteasome subunits β1, β2, and β5 was analyzed by Western blot analysis. **B**: I-45 and I-45-BTZ-R cells were treated with 40 nM bortezomib for 8, 24, or 30 hours. Proteasome chymotrypsin-like activity was determined by measuring the release of the fluorophore 7-amido-4-methylcoumarin (AMC) from the substrate N-succinyl-Leu-Val-Tyr-7 (LLVY) amido-4-methylcoumarin. Values are the mean ± SD of three experiments. * p < 0.05 as compared to untreated I-45 cells. **C**: I-45 and I-45-BTZ-R cells were treated with 40 nM bortezomib for 24, 48, or 72 hours. Expression of ubiquinated proteins were analyzed by Western blot analysis. **D**: I-45-BTZ-R cells were treated with 5, 10, or 20 μM AAF-CMK with or without 40 nM bortezomib for 48 hours. Cell viability was determined following treatment using the XTT assay. Control cells were treated with PBS and their viability was set as 100%. Values are the mean ± SD of triplicate assays from two experiments.

Several recent reports on acute myeloid leukemia, lymphoma, and MM described increased PSMB5 RNA and protein expression in bortezomib-adapted cells, and inhibition of PSMB5 expression partially restored bortezomib sensitivity in resistant cells, indicating that PSMB5 over expression is important for bortezomib resistance in cancer [16-19]. However, the present study did not detect a difference in PSMB5 protein expression between the two cell lines (Figure 2A). Moreover, expression of two other proteasome subunits, β1 (PSMB1) and β2 (PSMB2), which are responsible for caspase-like, or trypsin-like activity, respectively, was also not altered in the resistant cell line (Figure 2A).

Since bortezomib specifically inhibits proteasome chymotrypsin-like activity, we also evaluated proteasome chymotrypsin-like activity in parental and resistant cell lines using a substrate fluorescence assay. Although basal chymotrypsin-like activity was reduced approximately 30% in I-45-BTZ-R cells as compared to I-45 cells, both cell lines maintained approximately 25 to 40% of their original proteasome activity following bortezomib treatment (Figure 2B). These data indicated that chymotrypsin-like activity can be inhibited by bortezomib not only in parental I-45 cells, but also in I-45-BTZ-R cells, and that the mechanisms underlying I-45-BTZ-R cell resistance to bortezomib may be independent of proteasome activity.

Characteristic features of proteasome inhibition include the accumulation of ubiquitinated proteins, which can induce ER stress and UPR, and stabilization of pro-apoptotic genes, leading to mitochondrial membrane potential changes and apoptosis [11]. To this end, we evaluated the expression of ubiquitinated proteins in both cell lines following bortezomib treatment. As is shown in Figure 2C, basal levels of ubiquitinated proteins were similar in both of the cell lines. However, the concentration of ubiquitinated proteins in I-45 cells increased sharply 24 hours after exposure to 40 nM bortezomib, with further increases observed after 48 or 72 hours of drug exposure. Conversely, I-45-BTZ-R cells exhibited lower accumulation of ubiquitinated proteins after 24 hours of bortezomib exposure, and the concentration of ubiquitinated proteins returned to the basal level following 48 or 72 hours.

Since I-45-BTZ-R cells survived yet maintained the same low proteasome activity and showed much less accumulation of ubiquitinated proteins as did I-45 cells in response to toxic doses of bortezomib, we hypothesized that alternate cellular protease pathways might have become activated, essentially compensating for reduced 26S proteasome activity in I-45-BTZ-R cells and reducing bortezomib toxicity. One candidate protease was tripeptidyl peptidase II (TPPII), which plays a critical role in cleaving proteasomal-produced peptides into tripeptides prior to further degradation by other peptidases. TPPII upregulation has been reported in some lymphoma cells resistant to the proteasome inhibitor tri-leucine-vinyl-sulphone (NLVS), and inhibition of TPPII function using the specific inhibitor AAF-CMK either directly induces apoptosis or indirectly induces NLVS-mediated cell death [20,21]. In the present study, TPPII expression was not evident in either I-45 or I-45-BTZ-R cells by Western blot analysis (data not shown). Moreover, treatment with AAF-CMK at 5, 10, or 20 μM for 48 hours did not induce decreased I-45-BTZ-R cell viability (Figure 2D). Although combined treatment with AAF-CMK and bortezomib significantly decreased cell viability as compared with single agent treatment, cell viability remained very high (>85%). These results suggest that TPPII did not compensate for reduced proteasome activity in I-45-BTZ-R cells.

### Lack of cross-resistance to various anticancer drugs

**Figure 3:**
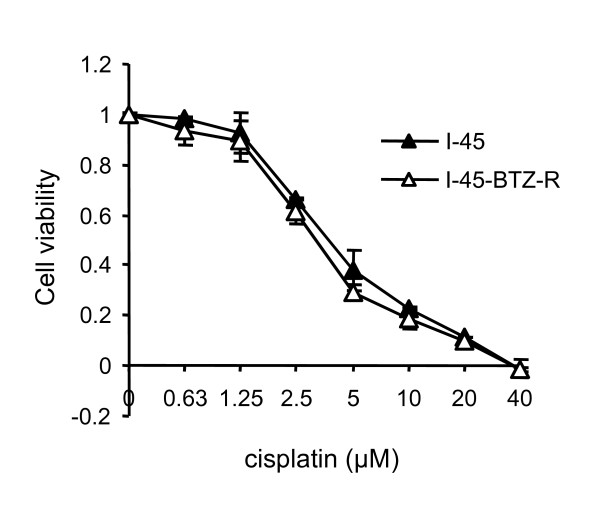
**Sensitivity of I-45 and I-45-BTZ-R cells to cisplatin**. I-45 and I-45-BTZ-R cells were treated with cisplatin (0.63 μM to 40 μM) for 72 hours. Cell viability was determined following treatment using the XTT assay. Control cells were treated with PBS and their viability was set as 100%. Values are the mean ± SD of triplicate assays from two experiments.

The cellular multi-drug resistance proteins MDR1 (p-glycoprotein) and MRP1 are critical transmembrane efflux pumps that transport various chemotherapeutic drugs out of cells. Both I-45 and I-45-BTZ-R cells showed no evidence of MDR1 or MRP1 by Western blot analysis (data not shown) and both cell lines demonstrated similar sensitivity to the chemotherapy drug cisplatin, which is commonly used to treat patients with mesothelioma (Figure 3). We also tested the responses of both cell lines to the chemotherapy drugs 5-fluorouracil and doxorubicin. As was observed with cisplatin, both cell lines showed similar sensitivity to treatment with 5-fluorouracil or doxorubicin (data not shown), indicating there the absence of cell line cross-resistance to common chemotherapy drugs.

### Bortezomib does not induce ER stress and UPR in I-45-BTZ-R cells

**Figure 4:**
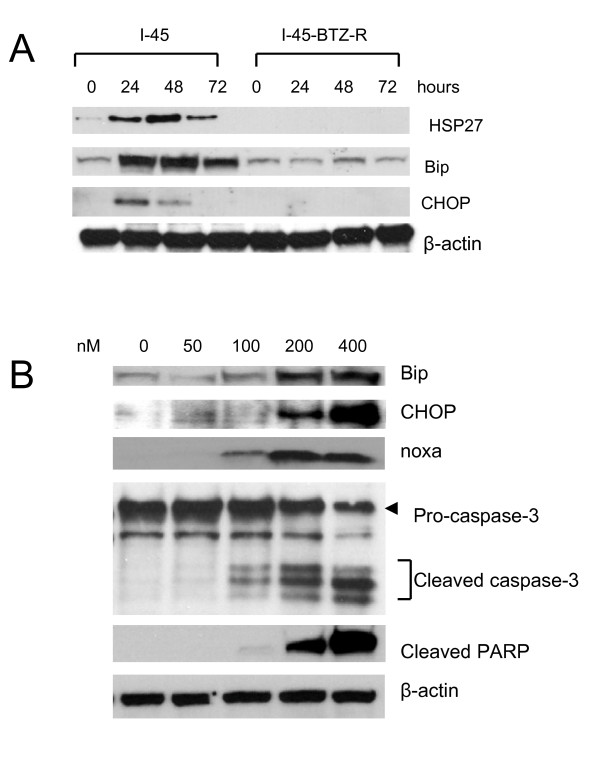
**ER stress activation and UPS induction**. **A**: I-45 and I-45-BTZ-R cells were treated with 40 nM bortezomib for 24, 48, or 72 hours. Expression of Bip, CHOP, or HSP27 was analyzed by Western blot analysis. **B**: I-45 and I-45-BTZ-R cells were treated with 50,100, 200, or 400 nM bortezomib for 48 hours. Expression of Bip, CHOP, or noxa, and cleavage of caspase-3 and PARP was analyzed by Western blot analysis.

Recent studies demonstrated that bortezomib activates ER stress and induces UPR in cancer cells [11,22]. Since the mitochondrial chaperone protein Bip and the pro-apoptotic transcriptional factor CHOP are markers of ER stress, we evaluated Bip and CHOP protein expression in I-45 and I-45-BTZ-R cells by Western blot analysis. Results indicated that basal Bip expression was similar in both cell lines. However, treatment with 40 nM bortezomib stimulated higher Bip and CHOP protein expression in I-45 cells but not in I-45-BTZ-R cells (Figure 4A). These results indicated that bortezomib treatment did not induce ER stress or UPR in I-45-BTZ-R cells. To determine whether lethal concentrations of bortezomib could induce ER stress and UPR, I-45-BTZ-R cells were incubated with increasing concentrations of bortezomib (40, 100, 200, and 400 nM). As is shown in Figure 4B, bortezomib at 50 nM did not induce expression of either Bip or CHOP. However, bortezomib at 100 to 400 nM induced an increase in the expression of both Bip and CHOP in parallel with an increase in apoptosis, as evidenced by increased cleavage of capase-3 and PARP.

**Figure 5:**
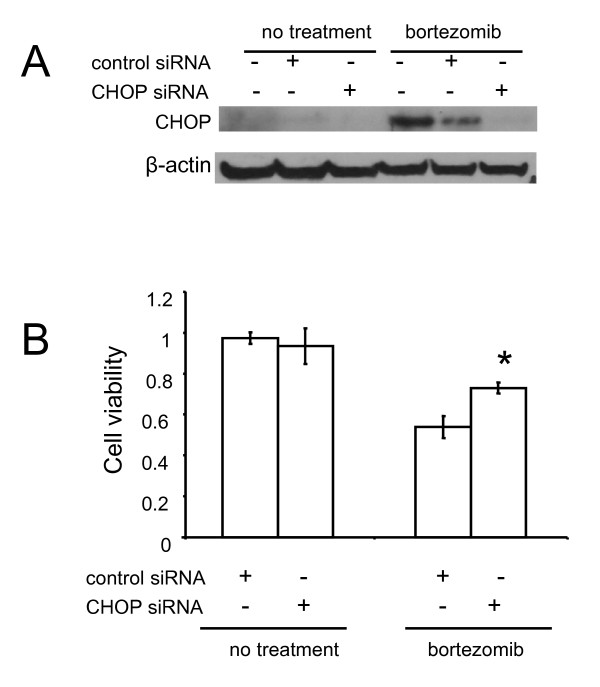
**Effects of siRNA inhibition of CHOP expression on bortezomib-induced changes in I-45-BTZ-R cell viability**. **A**: I-45-BTZ-R cells were transfected with 100 nM CHOP-specific siRNA or control siRNA. After 48 hours, cells were treated with 200 nM bortezomib for an additional 48 hours, and then CHOP protein expression was determined by Western blot analysis. **B**: I-45-BTZ-R cells were treated as described above, and then cell viability was determined using the XTT assay. Values are the mean ± SD of three experiments. * p < 0.05 as compared to treatment with bortezomib following transfection with control siRNA.

Since CHOP is a pro-apoptotic transcriptional factor during ER stress and UPR, we tested whether siRNA knockdown of CHOP expression could protect I-45-BTZ-R cells from bortezomib-induced cell death. Transfection with 100 nM of CHOP siRNAs for 48 hours followed by treatment with 200 nM bortezomib for an additional 72 hours effectively reduced bortezomib-mediated CHOP protein expression (Figure 5A). Knockdown of CHOP gene expression also protected I-45-BTZ-R cells from bortezomib-mediated apoptosis, as evidenced by a significant increase in cell viability from 53.9 ± 5.4% to 72.3 ± 2.6% in cells treated with in control siRNA versus CHOP-specific siRNA, respectively (Figure 5B). In addition, our data also demonstrated that siRNA-mediated knockdown of CHOP gene expression also partially protected parental I-45 cells from bortezomib-induced cell death (data not shown).

### Bortezomib induces noxa protein expression in I-45 cells but not in I-45-BTZ-R cells

**Figure 6:**
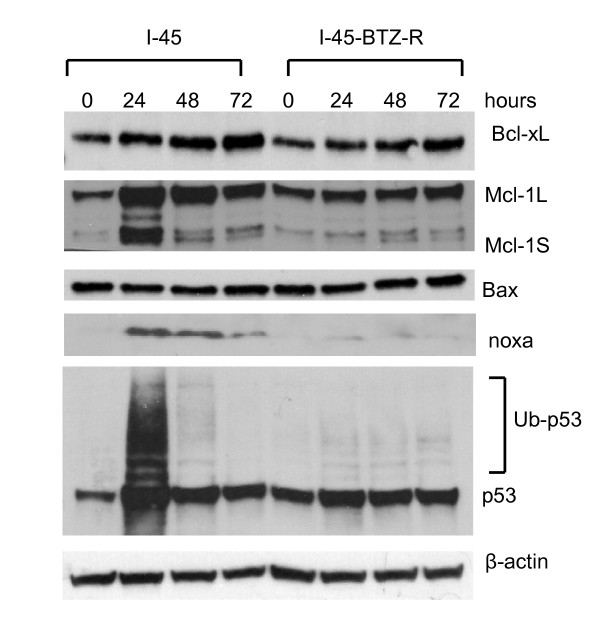
**Bcl-2 family protein expression following the bortezomib treatment**. I-45 and I-45-BTZ-R cells were treated with 40 nM bortezomib for 24, 48, or 72 hours. Expression of Bcl-xL, Mcl-1, Bax, noxa, or p53 was determined by Western blot analysis.

To better understand the molecular mechanisms underlying bortezomib resistance in I-45-BTZ-R cells, we examined BCl-2 family member protein expression by Western blot analysis. Results indicated that several Bcl-2 family proteins showed altered protein expression following bortezomib treatment (Figure 6). First, Bcl-x_L _protein expression increased slightly from 24 to 72 hours after treatment with 40 nM bortezomib in both of the cell lines, which may not account for bortezomib resistance in I-45-BTZ-R cells. Second, bortezomib treatment increased expression of the anti-apoptotic protein Mcl-1L in I-45 cells approximately 100% at 24, 48, or 72 hours, while expression of the pro-apoptotic protein Mcl-1S was increased approximately 150% at 24 hours, but then decreased at 48 and 72 hours, Pro-apoptotic Mcl-1S is reported to be a BH3 domain-only protein capable of dimerizing with Mcl-1L, thus reducing mcl-1L anti-apoptotic function [23]. Different results were observed in in I-45-BTZ-R cells, where bortezomib treatment slightly increased Mcl-1L expression over time, but no change in Mcl-1S expression was detected.

Strong expression of the noxa protein was also detected in I-45 cells after treatment with bortezomib, while noxa expression was barely detectable in I-45-BTZ-R cells subjected to the same treatment (Figure 6). Impressively, bortezomib at 100 to 400 nM also induced strong noxa expression in I-45-BTZ-R cells (Figure 4B), suggesting that noxa expression was correlated with bortezomib-induced cell death.

In addition, 40 nM bortezomib treatment induced strong p53 protein accumulation in parental I-45 cells, but only week p53 protein accumulation was observed in I-45-BTZ-R cells following bortezomib exposure (Figure 6).

## Discussion

In the present study, we developed a bortezomib-resistant cell line (I-45-BTZ-R) from a bortezomib-sensitive mesothelioma cell line (I-45). I-45-BTZ-R cells showed no cross-resistance with the common chemotherapeutic drugs cisplatin, 5-fluorouracil, and doxorubicin. Moreover, we observed that the bortezomib-adapted I-45-BTZ-R cells exhibited decreased growth kinemics as compared to the parental I-45 cells. We also found that I-45-BTZ-R cells did not over express the proteasome subunit PSMB5 as compared with the parental I-45 cells. In addition, 40 nM bortezomib induced similar inhibition of proteasome activity in the bortezomib-adapted cells and the parental I-45 cells, but significantly reduced accumulation of ubiquitinated protein accumulation. Further studies revealed that relatively low doses of bortezomib did not induce UPR in the bortezomib-adapted cells, while higher doses induced UPR with concomitant cell death, as evidenced by higher protein expression of the mitochondrial chaperone Bip and the ER stress-related pro-apoptotic gene CHOP. Bortezomib treatment of I-45-BTZ-R cells also failed to induce the accumulation of the pro-apoptotic genes p53, Mcl-1S, and noxa. These results suggest that evading UPR together with reduced induction of pro-apoptotic gene expression accounts for bortezomib resistance in these bortezomib-adapted mesothelioma cells.

Bortezomib is a reversible proteasome inhibitor that primarily targets the PSMB5 subunit, which is responsible for 26S proteasome chymotrypsin activity. Several research groups have recently developed bortezomib-resistant cells representing different type of cancer, including acute myeloid leukemia, lymphoma, and MM [16-19]. Most of the reported bortezomib-adapted cells have shown higher PSMB5 RNA and protein expression as compared to the respective parental bortezomib-sensitive cells. A missense point mutation has been reported in a highly conserved PSMB5 bortezomib-binding pocket and that siRNA-mediated reduction of PSMB5 expression restored bortezomib sensitivity in the bortezomib-resistant cell line [18]. In line with higher PSMB5 expression, most reported bortezomib-adapted cells showed increased proteasome activity as compared to their respective parental cell lines [16-19]. This increased activity has been used as a basis for explaining increased cell survival following a lethal challenge with proteasome inhibition. In the present study, over expression of proteasome subunit PSMB5 was not observed. We also did not find any mutations by DNA sequencing in the coding region of PSMB5 in both of the cell lines (data not shown). Accordingly, the bortezomib-adapted cells and I-45 cells showed the same degree of bortezomib-induced proteasome inhibition. I-45-BTZ-R cells showed much less accumulation of ubiquitinated proteins following bortezomib treatment. There are two possible explanations for this observation. First, an alternate protease pathway may compensate for reduced proteasome function. For example, in lymphoma, continuous inhibition of proteasome activity selected for proteasome inhibitor-resistant cells with lower proteasome activity, but higher expression of TPPII, which effectively replaced certain proteasome functions [20,21]. However, TPPII was not upregulated in I-45-BTZ-R cells (data not shown) and inhibition of TPPII activity using the specific inhibitor AAF-cmk had very little effect on the sensitivity of I-45-BTZ-R cells to bortezomib (Figure 2D). However, we could not exclude the possibility that the activity of other, as-yet unidentified proteases compensated for reduced proteasome function in I-45-BTZ-R cells. Second, the slower growth of I-45-BTZ-R cells may have induced a general decrease in protein synthesis, resulting in a reduction in the number of ubiquitinated proteins. This possibility may also partially explain our observation that relatively low concentrations of bortezomib did not induce ER stress and UPR in I-45-BTZ-R cells.

The ER is a eukaryotic organelle critical to the production and modification of one third of all cellular proteins. In the ER lumen, excessive accumulation of misfolded or oxidized proteins induced by ER stress leads to induction of the UPR, a protective mechanism that initially restores the luminal folding capacity of the ER, but will ultimately trigger cell death if the protective mechanism is overwhelmed. During ER stress, increased concentrations of protein chaperones, particularly Bip, can limit protein aggregation inside the ER and inhibit general protein synthesis in order to reduce the ER-Golgi network workload and the cellular damage induced by ER stress [22,24,25]. CHOP, another protein marker of ER stress, functions to mediate the execution of programmed cell death [22,24,25]. It has been reported that bortezomib induces apoptosis in MM [11] and head and neck squamous carcinoma cells [26] by activating ER stress concurrent with upregulation of Bip and CHOP. In the present study, we observed upregulation of Bip and CHOP in the bortezomib-sensitive cell line I-45 following bortezomib treatment, indicating bortezomib was able to induce ER stress in this mesothelioma cell line. However, low doses of bortezomib did not induce ER stress and apoptosis in the bortezomib-resistant cell line I-45-BTZ-R. Since proteasome inhibition resulting in the accumulation of ubiquitinated proteins is thought to induce ER stress and the UPR, reduced accumulation of ubiquitinated proteins in I-45-BTZ-R cells may have prevented ER stress induction and UPR-mediated cell death. Reduced accumulation of ubiquitinated proteins may have also decreased the stabilization of the pro-apoptotic proteins p53, Mcl-1S, and noxa in I-45-BTZ-R cells, thus further limiting bortezomib-induced apoptosis.

In agreement with the results of the present study, most other reported bortezomib-adapted cells did not exhibit much cross-resistance to most chemotherapeutic drugs [16,18]. These observations indicate that cancer cell resistance to bortezomib treatment can be overcome by most other therapies. Therefore, even though combination therapy using bortezomib together with chemotherapeutics does not show synergistic efficacy, such therapeutic approaches may still benefit patients through a reduction in the development of bortezomib resistance. Although expression of the multi-drug resistance proteins MDR1 and MRP1 was not detected in either I-45 or I-45-BTZ-R cell lines, we demonstrated that bortezomib can enter I-45-BTZ-R cells and access to the proteasome complex through direct assessment of proteasome activity. I-45-BTZ-R cells, which exhibit an approximately 30% decrease in basal proteasome activity as compared to I-45 cells, showed the same degree of proteasome inhibition following bortezomib treatment at doses that readily killed I-45-cells but spared I-45-BTZ-R cells.

## Conclusions

The present study provides evidence supporting a new mechanism of bortezomib resistance that is independent of proteasome subunit PSMB5 expression and proteasome activity. In I-45-BTZ-R cells, reduced accumulation of ubiquitinated proteins prevents bortezomib induction of ER stress and the UPR and reduces the stabilization of the pro-apoptotic proteins p53, Mcl-1S, and noxa. These changes may account for bortezomib resistance in this cell line.

## Methods

### Cell Culture and Reagents

The human sarcomatoid type mesothelioma cell line I-45 expressing wild type p53 was kindly provided by Dr. J. Testa (Fox Chase Institute, Philadelphia, PA). Cells were grown in RPMI 1640 medium supplemented with 10% fetal bovine serum, glutamine, and antibiotics. Cells were cultured at 37°C in a humidified incubator containing a 5% CO_2 _atmosphere. Bortezomib was kindly provided by Millennium: The Takeda Oncology Company (Cambridge, MA) and was dissolved in phosphate buffered saline (PBS) to make a stock concentration of 100 μM.

### Cell Viability Assay

Cells were seeded at a density of 5000 cells/well in 96-well plates one day before exposure to various treatments. Following treatment, cell viability was determined using an XTT cell viability assay (Cell Proliferation Kit II, Roche Molecular Biochemicals, Indianapolis, IN) according to the manufacturer's protocol and as previously described [27].

### Cell Growth Assay

Cells were seeded in 20 cm cell culture dishes at a density of 1 × 10^6 ^cells per dish. Cells were trypsinized at days 2, 4, 6, or 8 and stained with trypan blue. Viable cells were counted under a microscope using a hemocytometer.

### Western Blot Analysis

Western blot analysis was performed as described previously [27]. Rabbit polyclonal antibodies against PARP, caspase-3, Bcl-x_L_, Bax, Bip, HSP27, or CHOP were purchased from Cell Signaling (Beverly, MA). Rabbit polyclonal anti-human p53 and Mcl-1 antibodies were provided by Santa Cruz Biotechnology (Santa Cruz, CA). A mouse monoclonal anti-human noxa antibody was purchased from Calbiochem (San Diego, CA). Mouse monoclonal antibodies against human 20S proteasome subunits β1, β2, β5 and anti-ubiquitin (FK2H) antibodies were obtained from Biomol (Plymouth Meeting, PA).

### Small Interfering RNA Transfection

CHOP expression was silenced using a pool of four small interfering RNAs (siRNAs) directed against CHOP mRNA (Dharmacon, Lafayette, CO). Cells were transfected with 100 nM of CHOP-specific siRNA or siCONTROL Non-Targeting Pool siRNAs (Dharmacon) using the transfection reagent Dharma *FECT *1 (Dharmacon) according to the manufacturer's protocol. Cells were cultured for 48 hours, and then treated simultaneously with bortezomib for an additional 72 hours.

### Flow Cytometry

Cells were trypsinized, washed once with cold PBS, and then fixed with 70% ethanol overnight at 4°C. Fixed cells were suspended in PBS containing 25 μg/mL propidium iodide (Roche Diagnostics, Indianapolis, IN) and 10 μg/mL RNase A (Sigma-Aldrich, St. Louis, MO) at 3°C for 30 minutes. Flow cytometry analysis for cell cycle distribution and determination of the sub-G1 apoptotic cell population was performed as previously described [27].

### Proteasome chymotrypsin-like activity assay

Cells treated with bortezomib and untreated control cells were lysed in 20 mM Tris-HCl buffer (pH7.6) by repeated freezing in liquid nitrogen and thawing in a 37°C water bath. Cell lysate chymotrypsin-like activity was determined by measuring the release of the fluorophore 7-amido-4-methylcoumarin (AMC) from 10 μM of the substrate N-succinyl-Leu-Val-Tyr-7 (LLVY) amido-4-methylcoumarin (Sigma-Aldrich). Fluorescence was measured on a Flexstation microplate fluorometer (Molecular Devices, Sunnyvale, CA, USA) at excitation/emission wavelengths of 380/440 nm.

### Statistical Analysis

Differences among treatment groups were assessed by analysis of variance using PRISM 4 software. *P *values of = 0.05 were regarded as significant.

## List of Abbreviations

UPR: unfolded protein response; ER: endoplasmic reticulum; NFκB: Nuclear factor κB; MM: multiple myeloma; PSMB5: proteasome subunit β5; PSMB1: proteasome subunit β1; PSMB2: proteasome subunit β2; HSP27: heat shock protein 27; PBS: phosphate buffered saline; TPPII: tripeptidyl peptidase; NLVS: tri-leucine-vinyl-sulphone.

## Competing interests

The authors declare that they have no competing interests.

## Authors' contributions

LZ and WRS conceived the study, coordinated its design and execution, and drafted the manuscript. LZ, CP and YC performed the cell culture, cell viability assays, immunoblots and siRNA assays. XC and JEL were involved in the overall design of the study and helped draft the manuscript. All authors read and approved the final manuscript.
